# Impact of Calcium Lactate Concentration and Holding Time on Caviar-like Chicken Broth Hydrogel Beads

**DOI:** 10.3390/molecules30193926

**Published:** 2025-09-29

**Authors:** Betül Karslıoğlu, Eda Demirok Soncu, Tayyip Kızıldoğan, Dilan Gezer, Sıla Sudem Almaci

**Affiliations:** 1 Department of Gastronomy and Culinary Arts, Faculty of Tourism, Hasan Kalyoncu University, Gaziantep 27000, Turkey; 2 Department of Food Engineering, Faculty of Engineering, Ankara University, Ankara 06110, Turkey

**Keywords:** chicken broth, ionic gelation, alginate beads, molecular gastronomy, texture analysis, encapsulation efficiency

## Abstract

This study is the first to encapsulate chicken broth into caviar-like hydrogel beads (CBHBs) using ionic gelation, aiming to explore their potential in molecular gastronomy and functional food design. The effects of calcium lactate concentration (1%, 2.5%, and 5%) and post-gelation holding time (0, 30, and 60 min) on the physicochemical, morphological, mechanical, and sensory properties of chicken broth hydrogel beads were evaluated. The beads were produced by dropping a 1% sodium alginate–chicken broth mixture into calcium lactate solutions, followed by analysis of diameter, bulk density, pH, color, shape, texture, and consumer acceptance. Results revealed that higher calcium concentrations and extended holding times significantly decreased bead diameter and increased bulk density and hardness, indicating denser and more compact structures. Morphologically, increased calcium levels resulted in irregular, droplet-like shapes, with reduced sphericity. Instrumental color analysis showed higher a*, b*, and chroma values at higher calcium levels. Sensory evaluations demonstrated that samples with lower calcium concentrations and no post-gelation holding were significantly preferred by panelists in terms of softness and overall liking. These findings underscore the importance of optimizing calcium concentration and holding time in the design of alginate-based hydrogel beads and suggest that CBHBs have potential applications in molecular gastronomy and functional food product development.

## 1. Introduction

In recent years, molecular gastronomy, which has emerged from the intersection of food science and culinary arts, has enabled the development of products with novel taste, texture, and presentation characteristics by modifying food components at the molecular level. Within this scope, foods can be transformed into unusual forms such as spheres, foams, or sheets. Encapsulation, powdering, gelation, flavor/aroma transfer, sous-vide cooking, cryogenic processing with liquid nitrogen, smoking, and foaming are among the commonly applied techniques in molecular gastronomy [1]. Among these, hydrogel spherification is one of the most widely used methods [2]. This technique makes it possible to obtain high-quality products with desirable sensory properties and strong consumer appeal.

Hydrogel spherification is the process of converting liquids into edible spheres through gelation [3]. This is achieved via a controlled gelation mechanism based on the cross-linking reaction between sodium alginate and calcium chloride (CaCl_2_) [3,4]. The resulting spheres consist of a gel-like outer shell enclosing a liquid core, allowing liquids to be encapsulated within a solid structure. Sodium alginate, commonly used to form these gel structures, is a biodegradable, biocompatible, and non-toxic polysaccharide derived from brown seaweed [5]. It is widely utilized in the food industry as a gelling agent, thickener, and stabilizer [6,7]. The molecular structure of alginate is a linear binary copolymer composed of 1 → 4 linked α-D-mannuronic acid (M block) and β-L-guluronic acid (G block) residues [8]. In the presence of divalent ions such as Ca^2+^, Mg^2+^, Ba^2+^, and Sr^2+^, sodium alginate forms dense and strong gels [9]. Particularly, calcium salts trigger ion-exchange reactions that result in the formation of calcium alginate gel. The texture of this gel varies depending on the M/G ratio of the sodium alginate: low M/G ratios yield brittle and firm gels, whereas high M/G ratios lead to softer and more elastic textures [2,10]. Moreover, the choice of calcium salt (e.g., calcium lactate, calcium chloride, or calcium gluconate) strongly influences the gelation process and final product characteristics [2,11,12]. Calcium chloride is highly soluble and promotes very rapid gelation, but its use is limited in food applications due to the bitter taste it imparts [13]. Calcium gluconate, with low solubility, results in very slow gelation and allows fine-tuning of gel thickness, although it is less practical for large-scale processing [14]. Calcium lactate, on the other hand, offers a balanced alternative: it provides moderate gelation speed, avoids the bitterness associated with calcium chloride, and is organoleptically more acceptable [2,15,16]. For these reasons, calcium lactate was selected in this study as the calcium source for bead formation, in order to balance gelation kinetics with consumer acceptability.

Spherification can be carried out using two main techniques: basic spherification and reverse spherification. In basic spherification, a liquid mixed with sodium alginate is dropped into a calcium-containing solution; in reverse spherification, a calcium-containing liquid is dropped into a sodium alginate solution [17]. In both methods, the resulting spheres consist of a gel membrane enclosing a liquid center, creating products that provide a “bursting” effect in the mouth in terms of both taste and texture. This calcium alginate gel, in spherical form, is referred to by different names such as caviar, ravioli, or beads, depending on its size and application [18]. In this study, these structures are referred to as caviar-like hydrogel beads. The literature reports that caviar-like products have been developed using the spherification technique from various liquid bases, such as seasoned soy-based sauces (mentsuyu), coffee extracts, and melanin-free squid ink [19,20,21]. In addition, the alginate spherification method has also been used to produce egg analogs or faux caviars as substitutes for fish roes, including those of carp, sturgeon, and flying fish [22,23,24]. However, to the best of our knowledge, no study has examined the encapsulation of meat-based broths, particularly chicken broth, using this technique. This gap highlights the novelty of the present work.

Chicken broth is a valuable food component that is widely used in traditional cuisines and is notable for its rich nutritional profile. Due to its characteristic flavor and aroma, it is highly appreciated as a culinary ingredient worldwide [25,26]. It is commonly utilized as a versatile liquid flavoring agent in soups, pilafs, and various Turkish dishes, particularly in sauce preparation. However, its liquid form presents certain limitations in terms of shelf life, portability, and convenience of use. To overcome these limitations, the development of alternative carrier systems may enable more stable and functional applications of chicken broth across different food matrices.

To the best of our knowledge, this is the first study to investigate caviar-like chicken broth hydrogel beads (CBHBs) prepared using ionic gelation with calcium alginate-based systems. Additionally, it is the first to simultaneously evaluate the effects of calcium lactate concentration (1%, 2.5%, and 5%) and post-gelation holding time (0, 30, and 60 min) on the physicochemical, textural, and sensory properties of the hydrogel beads. In this regard, the study aims to contribute both to formulation development and to molecular gastronomy–based product designs within the scope of functional food applications.

## 2. Results and Discussion

### 2.1. Effect of Calcium Lactate Concentration and Holding Time on Physicochemical Properties of CBHBs

The average diameter (d_l_), pH, and bulk density values of the beads varied depending on calcium lactate concentration and post-gelation holding time (Table 1). The diameters of the beads ranged between 2.25 and 2.95 mm, with a clear decrease observed as calcium lactate concentration increased (*p* < 0.001). In particular, the groups prepared with 5% calcium lactate exhibited smaller bead diameters compared to the other groups. This can be explained by the formation of a firmer gel shell compressing the inner matrix, leading to water loss from the gel, known as syneresis. Syneresis is a characteristic property of gel structures and occurs when the gel network, which retains water through hydrogen bonding, contracts over time and expels water into the outer phase via diffusion [27]. The observed water loss caused the gel structure to contract in volume, resulting in a smaller and more compact morphology. Additionally, holding time had a statistically significant effect on bead diameter (*p* < 0.001); the diameters measured at 60 min were significantly lower than those recorded at 0 and 30 min. This reduction in size may be attributed to increased cross-linking within the gel matrix over time, which likely leads to a denser internal network and subsequent shrinkage. Consistent with these findings, a previous study on caviar-like beads containing melanin-free squid ink reported that the diameter of beads with 3% NaCl ranged from approximately 2.68 to 2.89 mm, while those with 5% NaCl ranged from 3.27 to 3.43 mm [19]. Similarly, a study on alginate/cocoa beads reported that increasing calcium concentrations led to a significant reduction in bead diameter [28].

The pH values of the beads were significantly affected by the interaction effect of calcium lactate concentration and holding time factors (*p* < 0.001). This indicates that the pH response among the groups with the same calcium lactate concentration increased with increasing holding time. Additionally, higher pH values were measured in calcium lactate concentrations of 2.5% and 5% as compared to that of 1% in the same holding time (*p* < 0.001). In addition to these evaluations, when all groups were compared, the lowest pH value was observed in the C1T0 group (6.59 ± 0.05), while the highest was recorded in the C5T60 group (6.96 ± 0.03). These findings suggest that elevated calcium content and prolonged holding time collectively influence the internal ionic balance and buffering capacity of the beads. According to the literature, gels produced at pH ≥ 5 exhibit lower microstructural connectivity and density, which facilitates syneresis and shrinkage. [8]. Therefore, disruptions in ionic balance may have contributed to changes in the internal pH of the capsules which supports lower density values at elevated lactate concentration and extended holding time. Several studies have also reported that the microstructural properties of alginate beads are strongly influenced by the pH during synthesis [8,29,30]. In contrast to our findings, Yuasa, Tagawa and Tominaga [20] reported pH values ranging from 4.8 to 5.0 in their mentsuyu caviar capsules prepared using the spherification technique.

Bulk density is widely used as an indicator of the compactness and structural integrity of particulate or encapsulated systems, providing insights into internal packing efficiency and porosity [31]. In the present study, the bulk density of the samples ranged from 0.535 to 0.641 g/mL. A statistically significant decrease in bulk density was observed with increasing calcium lactate concentration (*p* < 0.001), whereas the main effect of holding time was not statistically significant. The highest bulk density was measured in beads prepared with 1% calcium lactate concentration. This could be attributed to their relatively lower porosity and more compact structure, while the smaller diameters observed at higher calcium levels were accompanied by increased porosity, which reduced bulk density [32]. At higher calcium levels, more intensive cross-linking promoted the formation of a firmer outer shell, but simultaneously increased porosity in the internal structure, which may have reduced bulk density [33]. Similar explanations have been reported in the literature, where the macroporous structure of alginate matrices was associated with lower bulk density values [34]. Furthermore, the hydrophilic or lipophilic character of the encapsulated core material has also been shown to influence the moisture content and density of hydrogels [34,35]. In a study by Aykın-Dinçer, et al. [36], who encapsulated sage extract at varying concentrations, bulk density values were reported to range between 0.784 and 0.917 g/mL. Similarly, another study reported a bulk density value of 0.54 g/mL in alginate microcapsules produced from sour cherry by-products, supporting the relatively low bulk density values observed in the present study [34].

### 2.2. The Optic Images and Shape Classification of CBHBs

**Figure 1:**
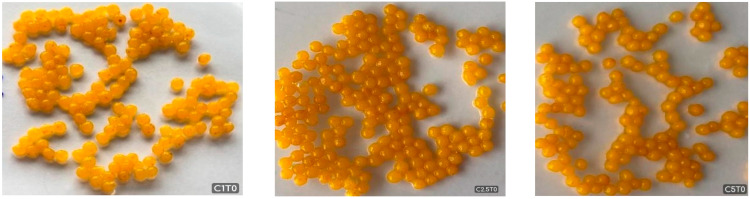
Optical images of CBHB samples.

Optical images of bead samples prepared with different calcium lactate concentrations (1%, 2.5%, and 5%) are presented in Figure 1. Based on morphological descriptors previously defined by researchers [36,37], each bead could be classified into one of four categories: spherical, droplet, pear-shaped, or egg-shaped.

In this study, various dimensionless shape indicators were evaluated to assess the morphological characteristics of the beads (Table 2). It was observed that circularity and aspect ratio were insufficient in fully reflecting the deformation of the droplets. In contrast, the sphericity factor (SF) was found to be more sensitive and effective in capturing shape changes, and therefore, it was adopted as the primary indicator in this study. According to the literature, beads with a sphericity factor below 0.05 are considered spherical. This is because the extent of deformation could not be obviously differentiated by human vision.

The analysis revealed that only the C1 group exhibited a spherical shape, while all other groups showed clear signs of deformation. This indicates that increasing calcium lactate concentration reduces sphericity and contributes to the formation of irregular shapes. These findings emphasize the crucial role of gelation dynamics and interfacial tension between the alginate solution and the calcium bath in shaping the final bead morphology [38]. As stated in the previous study, higher calcium levels were associated with asymmetrical and elongated structures, likely due to the formation of a rigid gel shell on the droplet surface that disrupts the retention of a uniform spherical form [39]. The prevalence of droplet-like beads at elevated calcium concentrations may be further explained by insufficient interfacial stabilization and gravitational elongation during bead formation, both of which contribute to morphological asymmetry [19,21]. Alginate concentration is another key parameter influencing bead morphology and size distribution, as it directly affects droplet formation and gelation behavior. Chan et al. [37] reported that alginate concentrations below 1% result in nonspherical, irregular capsules during extrusion, likely due to a lack of sufficient carboxyl groups required for effective cross-linking. This finding is in agreement with the deformation patterns observed under low calcium concentrations in the present study. Similar morphological distortions have been reported in studies examining the effects of gelling ion concentration and cross-linking kinetics.

Our results are also in line with the findings of Kaltsa et al. [40], who encapsulated Moringa oleifera extract in calcium alginate chocolate beads. In their study, rapid gelation at high calcium ion levels led to the development of a rigid outer shell, resulting in flattened and irregular bead shapes. The dominance of tear-shaped beads observed in our samples can be attributed to similar mechanisms. Both studies concluded that high calcium ion concentrations promote rapid shell formation, leading to shape distortion. Moreover, inadequate interfacial stabilization and gravitational effects further contributed to asymmetric morphologies [6,37,41,42]. Overall, these findings underscore the importance of controlling cross-linking conditions and optimizing ion concentrations to achieve uniform and stable bead morphology.

These morphological irregularities may also have practical implications. From a consumer perspective, visual appearance plays a critical role in product acceptance, and irregular or elongated beads may be perceived as less appealing compared to uniformly spherical ones. This observation is consistent with our sensory evaluation results, where samples prepared with lower calcium concentrations that maintained more spherical shapes were more preferred by panelists in terms of softness and overall liking. In culinary applications, irregular shapes may affect uniformity during cooking or plating, thereby influencing texture consistency and overall presentation. Therefore, controlling bead morphology is important not only for structural stability but also for consumer acceptance and practical use in gastronomy.

### 2.3. Effect of Calcium Lactate Concentration and Holding Time on Color Properties of CBHBs

Color is one of the key quality parameters that influence consumer acceptance and purchasing behavior, and it also plays a decisive role in other important sensory attributes [40]. According to the data obtained in this study, the L* values of the CBHB samples ranged from 47.82 to 51.91, a* values from 2.46 to 5.35, b* values from 61.47 to 65.35, and Chroma values from 61.52 to 65.57 (Table 3). The findings indicate that calcium lactate concentration caused statistically significant differences in instrumental color values (*p* < 0.001). Specifically, as the calcium concentration increased, a decrease in L* values and an increase in a*, b* and chroma values were observed. This can be explained by the formation of a denser and more opaque structure in the gel matrix due to the increased ionic density. Additionally, the stronger retention of yellowish pigments derived from ginger, present in the chicken broth, within this structure may further support these changes. From a consumer perspective, such color changes may be critical. Lower L* values (darker appearance) and higher b* values (more yellowish tones) may lead the beads to appear less fresh and less visually appealing, potentially reducing consumer acceptance. This interpretation is consistent with our sensory evaluation results, where samples with lower calcium concentrations (brighter and less yellow) received higher overall liking scores.

Similarly, various studies in the literature have reported changes in color parameters depending on the type of sample and formulation used. For instance, in alginate beads prepared with melanin-free squid ink, greenish (−a*) and bluish (−b*) tones increased, which was accompanied by a decrease in L* values [19]. Likewise, in coffee extract-based hydrogel beads prepared with varying concentrations of sodium alginate, increasing alginate concentration led to an increase in L* values, a decrease in a* values, and an increase in b* values [21].

### 2.4. Effect of Calcium Lactate Concentration and Holding Time on Mechanical Properties of CBHBs

Based on the compression test of Texture Profile Analysis (TPA), various mechanical texture parameters were measured in chicken beads (Table 4). In CBHB samples prepared using ionic gelation, the interaction between calcium concentration and post-gelation holding time was found to be statistically significant in only hardness values (*p* < 0.001). Hardness degree increased with increasing holding time among groups prepared using the same lactate concentration (1% and 2.5%) and, specifically, the increase at 30 min. was statistically important (*p* < 0.001). The evaluation from another factor showed that increasing calcium lactate concentration resulted in the formation of harder beads at only initial stage (0 min.). These findings have asserted that higher calcium concentrations lead to a more cross-linked gel network, thereby increasing the hardness and cross-linking continues during the post-gelation period [28].

Springiness refers to the ability of a sample to return to its original shape after the removal of an external force. In CBHB samples, the main effect of calcium lactate concentration was found to be significant (*p* < 0.05). The springiness value was statistically lower in beads prepared with 5% calcium lactate as compared to other concentrations. This finding shows that higher calcium levels result in a firmer and tighter gel shell structure, significantly affecting the deformation recovery capacity of the beads (*p* < 0.05). Known as the elasticity index, this textural parameter is related to the degree of relaxation and ranges from 0 to 1. A high springiness value indicates a “rubbery” texture, whereas a low value indicates a “brittle” structure [28]. Accordingly, beads in the C5 group exhibited lower elasticity, reflecting a more rigid structure, while higher springiness observed in the C1 group suggests that lower Ca^2+^ concentrations enable the formation of more flexible and recoverable gel matrices. Overall, CBHBs produced with 1% calcium exhibited higher elasticity, indicating a more rubber-like texture. Moreover, holding time did not have a statistically significant effect on springiness (*p* > 0.05), suggesting that elasticity was mainly influenced by calcium concentration rather than storage duration.

Cohesiveness, another textural parameter determined by TPA, is associated with the strength of internal bonds within the product [43]. No statistically significant differences were determined among beads, although cohesiveness values decreased with increasing calcium lactate concentration. This finding may be explained by the increasing syneresis in highly cross-linked gels with higher ionic content and lactate concentration, leading to structural weakening over time. Gumminess results showed significant differences in CBHB samples with respect to the main effects of both calcium lactate concentration (*p* < 0.001) and holding time (*p* < 0.001). CBHBs prepared with lower calcium concentration (1%) exhibited significantly higher gumminess values compared to those with higher calcium content. Gumminess is related to the energy required to disintegrate the hydrogel beads [43]. In line with this statement, it could be noted that CBHBs containing 1% calcium lactate required more energy to be broken down than those prepared with 5% calcium. On the other side, extended holding time significantly decreased gumminess values in bead samples.

Chewiness is a critical parameter that directly affects the chewability and sensory acceptability of caviar-like hydrogel beads. In CBHB samples, chewiness values significantly decreased with increasing calcium lactate concentration and post-gelation holding time as a main effect of each factor. The highest chewiness value was observed in the C1 group at initial time, indicating that these beads had a more resilient texture and remained longer in the mouth during mastication. From a culinary perspective, excessive chewiness reduces the resemblance to real caviar, which is characterized by a delicate burst-in-the-mouth sensation. This helps explain why beads with lower chewiness were more preferred in sensory evaluation, as they provided a texture closer to consumer expectations of caviar-like products.

### 2.5. Effect of Calcium Lactate Concentration and Holding Time on Sensorial Properties of CBHBs

**Figure 2:**
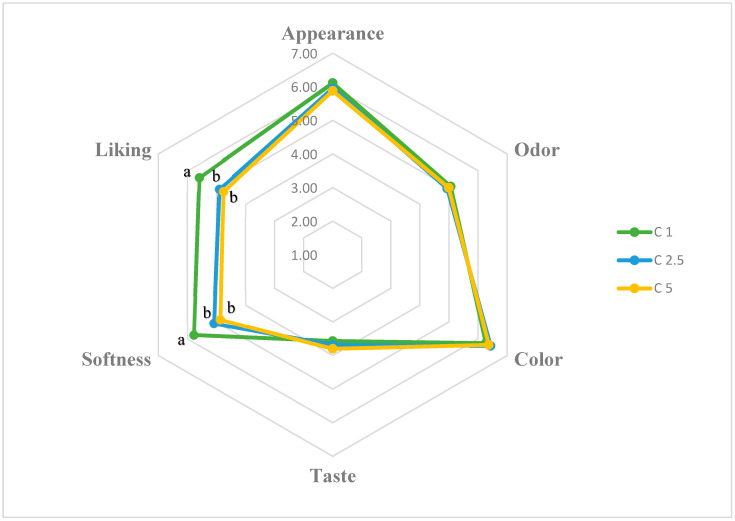
Effect of calcium concentration on the sensory properties of CBHB samples. Data are presented as mean values (*n* = 29). a, b: Means followed by different letters are significantly different (*p* < 0.05).

The effects of calcium concentration on the sensory properties of CBHBs (appearance, odor, color, taste, softness, and overall liking) are presented in Figure 2. The mean ± SEM scores for these attributes were as follows: appearance (6.12 ± 0.02, 5.95 ± 0.03, and 5.88 ± 0.07 for C1, C2.5, and C5, respectively), odor (5.05 ± 0.04, 4.94 ± 0.02, and 4.98 ± 0.06), color (6.27 ± 0.06, 6.43 ± 0.02, and 6.37 ± 0.02), taste (3.56 ± 0.04, 3.69 ± 0.06, and 3.80 ± 0.09), softness (5.78 ± 0.02, 5.08 ± 0.05, and 4.87 ± 0.04), and overall liking (5.58 ± 0.04, 4.90 ± 0.09, and 4.76 ± 0.11).

Among these parameters, only softness and overall liking differed significantly among the samples (*p* < 0.05). These results indicate that higher calcium concentrations decreased both softness and overall acceptability of the beads, likely due to increased firmness and reduced water content. In contrast, appearance, odor, color, and taste did not differ significantly (*p* > 0.05), suggesting that these attributes were not substantially influenced by calcium concentration or did not change to a perceptible degree. Furthermore, correlation analysis revealed a strong negative relationship between instrumental hardness and sensory softness (r = −0.921, *p* < 0.05), confirming that samples with higher instrumental hardness were consistently perceived as less soft by the panelists.

The low taste scores observed in the study were likely due to panelists expecting a pronounced chicken flavor from the beads, which was not fully delivered because of the taste-masking effect of the applied microencapsulation technique. However, this outcome may be considered an advantage for consumers who dislike or are skeptical of chicken broth, as the mild flavor may increase its acceptability among such individuals. Our finding is consistent with previous literature suggesting that the barrier properties of the gel matrix limit aroma release [44]. In addition to the hedonic evaluation, a ranking test was conducted to determine the panelists’ preferences for the C1, C2.5, and C5 samples immediately after preparation (0 min). All of the panelists (100%) ranked the C1 group first, while 93% ranked the C2.5 group second, and 79% preferred the C5 group in the third place. Overall, the data demonstrate that calcium lactate concentration plays critical roles, primarily affecting texture-related attributes such as softness and overall liking, in shaping the sensory quality of CBHBs.

## 3. Materials and Methods

### 3.1. Experimental Location and Ethical Aspects

The experiment was carried out at the Department of Food Engineering, Faculty of Engineering, Ankara University, Türkiye. This study was approved by the Research Ethics Committee of Hasan Kalyoncu University (Protocol number: E-97105791-050.04-70865).

### 3.2. Materials, Chemicals and Apparatus

In the production of chicken beads, pasteurized chicken broth (Fide, İstanbul, Turkey) obtained from a local market was used. The chicken broth used (95%) contained carrot, celery stalk, onion, garlic, apple vinegar, whole black pepper, thyme, bay leaf, ginger, and clove. Sodium alginate (M/G ratio = 1.3; Tito, Ankara, Turkey) was used as the coating material, and calcium lactate (Merck, Darmstadt, Germany) was used as the gelling (hardening) solution.

### 3.3. Preparation of CBHB

The production of caviar-like chicken broth hydrogel beads (CBHB) was carried out by applying minor modifications to the method described by Yuasa et al. [20]. Sodium alginate (1 g) was added to chicken broth (99 g) preheated to 90 °C and homogenized using a homogenizer (Model Miccra D9, Mühlheim am Main, Germany) for 5 min at maximum speed. The final mixture contained 1% (*w*/*w*) sodium alginate and 99% (*w*/*w*) chicken broth, and was cooled at 4 °C for 3 h prior to use. At this stage, calcium lactate solutions at three different concentrations (1%, 2.5%, and 5%) were prepared in distilled water at room temperature, and the pH of all solutions was adjusted to 7 using 0.1 N citric acid and/or 0.1 N NaOH to ensure consistent gelation conditions.

**Figure 3:**
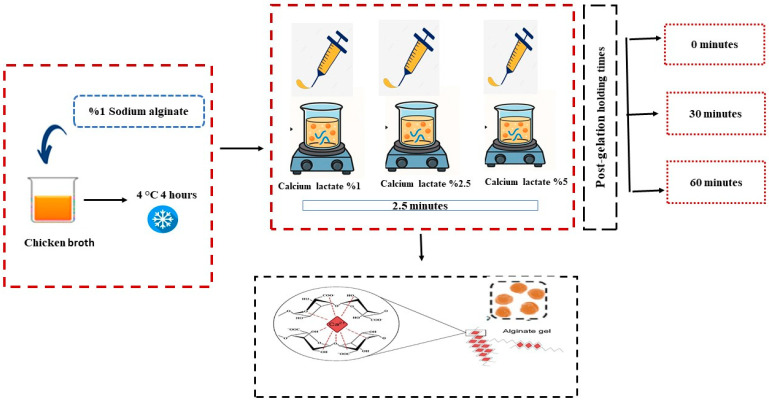
Process of preparing caviar-like chicken broth hydrogel beads by ionic gelation at different calcium lactate concentrations and post-gelation holding times.

The chicken broth solutions were extruded using a molecular caviar kit and dripped into calcium lactate baths of varying concentrations to obtain CBHBs. The tip of the caviar dropper was fixed 5 cm above the surface of the gelling bath. To prevent the beads from sticking to each other, the gelling solution was stirred gently with a magnetic stirrer set at low speed throughout the gelation period. Drops were added in sets of ten, and the beads were left for 2.5 min, which was defined as the gelation time. The calcium lactate solution was refreshed after every 50 drops were prepared. The solidified CBHBs were collected using a strainer, rinsed with distilled water to remove excess Ca^2+^ ions, and then drained before storage. The beads were stored at 4 °C for different durations (0, 30, and 60 min) to evaluate the effect of post-gelation holding time. The sample preparation process is illustrated in Figure 3, and the sample codes with corresponding conditions are presented in Table 5. For each experimental condition, approximately 200 beads were produced. All samples were prepared and analyzed at least in duplicate.

### 3.4. Analyses

#### 3.4.1. pH Measurements

The pH values of CBHB samples were measured using a digital pH meter (Ohaus Starter 2100, Parsippany, NJ, USA). For this purpose, 10 g of each sample was homogenized in 100 mL of distilled water using an Ultraturrax homogenizer (Model Miccra D9, Mühlheim am Main, Germany) at approximately 50 rpm for 2 min to ensure uniform dispersion prior to measurement.

#### 3.4.2. The Shape Images and Bead Size Measurements

The particle size of the beads was estimated by means of image analysis. Photographs of the bead samples were taken using a digital camera (Nikon COOLPIX P1000, Nikon Corporation, Tokyo, Japan) and transferred to a computer. To calculate the geometric properties of the beads, the freely available image analysis software ImageJ v.1.46r was used, and the calculations were performed using Microsoft Excel. All beads were thoroughly mixed, and 30 beads were randomly selected for image analysis. The calculated parameters were area (mm^2^), perimeter (mm), and maximum and minimum Feret diameters (mm). The shape of the CBHBs was quantitatively evaluated using the dimensionless shape indicators defined in Table 6.

#### 3.4.3. Bulk Density Analysis

The bulk density of the samples was determined using the method reported by Aykın-Dinçer et al. [36]. For this purpose, 5 g of the sample was placed into a 25 mL graduated cylinder, and the volume occupied by the sample was recorded. Bulk density was calculated by dividing the sample mass by the measured volume and expressed as g/mL.

#### 3.4.4. Color Measurement

A randomly selected CBHB sample was placed on the colorimeter probe, and the surface color was evaluated using a Minolta colorimeter (Chromameter CR-300, Minolta Camera, Osaka, Japan), which was calibrated with a white calibration plate (Reference number: 1353123; Y = 92.70; x = 0.3133; y = 0.3193). Color measurements were performed according to the CIELAB color system, recording lightness (L*; black/white), redness (a*; green/red), and yellowness (b*; blue/yellow) values. The samples were placed on a white background, and measurements were taken from four different points on each sample.

#### 3.4.5. Mechanical Properties

To determine the textural properties of CBHB, the method was applied with minor modifications [19].

Measurements were carried out using a texture analyzer (Texture Exponent 32, Stable Micro Systems, Godalming, Surrey, UK). The analysis was performed in a glass Petri dish (60 mm diameter × 15 mm height, Superior) completely filled with CBHB, using a P25 cylindrical probe at a speed of 0.5 mm/s. From the texture profile analysis (TPA) curves, mechanical parameters such as hardness, cohesiveness, and elasticity were directly measured, while secondary parameters such as gumminess and chewiness were calculated using the device software. To investigate the effects of calcium lactate concentration and post-gelation holding time on the textural properties of CBHB, a 20% compression was applied to avoid damaging the beads. To obtain representative results, samples were prepared in four separate Petri dishes for each analysis, a single measurement was taken from each dish, and the average of the values was reported.

#### 3.4.6. Sensory Analysis

A sensory evaluation of the samples was conducted as a consumer panel with 29 semi-trained volunteers, and informed consent was obtained from all participants in accordance with Hasan Kalyoncu University’s ethical procedures. The panel consisted of individuals aged between 25 and 55 years (21 females and 8 males). Exclusion criteria included smoking, symptoms of cough or flu, and the use of prescribed medications. The evaluations were conducted in a room with natural daylight and at ambient temperature. The samples were presented using a blind testing method, coded with three-digit random numbers, and served in transparent containers. Water and grissini were provided for palate cleansing between samples. Panelists were asked to evaluate appearance, odor, color, taste, softness, and overall liking using a 9-point hedonic scale (1: extremely dislike, 5: neither like nor dislike, 9: extremely like) [45]. In addition to the hedonic evaluation, a ranking test was performed to assess the panelists’ preference for the chicken beads prepared with three different calcium lactate concentration at only initial time (0 min).

#### 3.4.7. Statistical Analysis

For statistical analysis, the experiments were conducted in two independent replicates at different times, and all parameters were measured in duplicate. The mean values of each parameter analyzed in CBHBs were evaluated using analysis of variance (ANOVA) in Minitab statistical software (Version 22.0, Minitab Inc., Enterprise Drive, State College, PA, USA). A two-way ANOVA design was employed, with calcium lactate concentration and post-gelation holding time as independent variables. When the interaction between calcium lactate concentration and holding time was not significant, the main effects of calcium lactate concentration or holding time were evaluated to determine the differences between groups. This design enabled the assessment of both main and interaction effects. In addition, Pearson correlation analysis was performed to evaluate the relationship between instrumental hardness and sensory softness. When the *p*-value was less than 0.05, Tukey’s post hoc test was applied to determine significant differences. Results were reported as mean ± SEM (standard error of the mean).

## 4. Conclusions

This study is the first to investigate the encapsulation of chicken broth into caviar-like hydrogel beads using calcium alginate systems and to examine how varying calcium lactate concentrations and post-gelation holding times affect their structural and sensory characteristics. The results demonstrated that both parameters significantly influence the physicochemical, mechanical, and morphological properties of the beads. Higher calcium concentrations resulted in firmer, smaller, and more deformed beads due to enhanced cross-linking and syneresis, while prolonged holding times further contributed to shrinkage and increased hardness. From a sensory standpoint, samples with lower calcium concentrations and shorter holding durations were more favorably evaluated, especially in terms of softness and overall acceptability. In particular, the optimum condition for producing caviar-like chicken broth hydrogel beads was achieved at 1% calcium lactate concentration with no post-gelation holding (0 min), which provided the best balance of texture and consumer acceptability. These outcomes highlight the critical role of formulation and processing parameters in achieving desirable texture and consumer satisfaction. The findings offer valuable insights for the application of molecular gastronomy techniques in developing novel, stable, and sensorially appealing functional food products using broth-based matrices. Future research should address the encapsulation of other broths and flavors, the release kinetics of volatiles, the incorporation of bioactive compounds, and scale-up processing approaches. Such directions will further establish caviar-like hydrogel beads as versatile carriers for innovative, nutritious, and sensorially attractive food products.

### Implications for Research and Practice

This study has certain limitations that should be acknowledged. Sensory evaluation was conducted with a semi-trained consumer panel, which may not provide the same level of discrimination as fully trained sensory panels. Although the panelists were guided according to standard sensory practices, their evaluations could be influenced by subjective variability. Furthermore, the novelty effect of caviar-like chicken broth beads may have introduced potential bias in preference ratings, as unfamiliar product forms can sometimes enhance or reduce consumer acceptance independent of intrinsic quality. Future studies should incorporate larger and more diverse panels, including both trained assessors and general consumers, to validate and broaden the sensory findings.

## Figures and Tables

**Table 1:** Physicochemical Properties of CBHB Samples *.

	Interaction Effect (Calcium Lactate Concentration × Holding Time)		
CBHB Samples	d_l_ (mm)	pH	Bulk Density (g/mL)
C1T0	2.95 ± 0.11	6.59 ± 0.05 ^z2^	0.607 ± 0.02
C1T30	2.82 ± 0.09	6.76 ± 0.45 ^y2^	0.618 ± 0.02
C1T60	2.75 ± 0.12	6.93 ± 0.05 ^x1^	0.641 ± 0.02
C2.5T0	2.50 ± 0.32	6.83 ± 0.03 ^x1^	0.535 ± 0.02
C2.5T30	2.45 ± 0.18	6.88 ± 0.06 ^x1^	0.547 ± 0.02
C2.5T60	2.36 ± 0.31	6.90 ± 0.09 ^x1^	0.550 ± 0.03
C5T0	2.36 ± 0.45	6.87 ± 0.06 ^y1^	0.576 ± 0.05
C5T30	2.32 ± 0.81	6.90 ± 0.05 ^xy1^	0.546 ± 0.01
C5T60	2.25 ± 0.52	6.96 ± 0.03 ^x1^	0.541 ± 0.02
	**Main effects**		
	**Holding time**		
	**0.**	**30.**	**60.**
**d_l_ (mm)**	2.57 ± 0.12 ^A^	2.51 ± 0.12 ^A^	2.37 ± 0.14 ^B^
**pH**	6.76 ± 0.06	6.85 ± 0.03	6.93 ± 0.02
**Bulk density**	0.572 ± 0.02	0.570 ± 0.02	0.577 ± 0.02
	**Main effects**		
	**Calcium lactate concentration**		
	**C1**	**C2.5**	**C5**
**d_l_ (mm)**	2.87 ± 0.02 ^a^	2.39 ± 0.05 ^b^	2.20 ± 0.05 ^c^
**pH**	6.76 ± 0.06	6.87 ± 0.06	6.91 ± 0.06
**Bulk density**	0.622 ± 0.01 ^a^	0.544 ± 0.01 ^b^	0.554 ± 0.02 ^b^
	**Calcium lactate concentration × Holding time**	**Holding time**	**Calcium lactate concentration**
**d_l_ (mm)**	ns	*p* < 0.001	*p* < 0.001
**pH**	*p* < 0.001	*p* < 0.001	*p* < 0.001
**Bulk density**	ns	ns	*p* < 0.001

* Mean values ± standard error of the mean; ns: not significant. a, b, c: Values with different letters (in the same line) indicate statistically significant differences due to the main effect of calcium lactate concentration (*p* < 0.001). A, B: Values with different letters (in the same line) indicate statistically significant differences due to the main effect of post-gelation holding time (*p* < 0.001). x, y, z: Values with different letters (in the same row) within the same calcium lactate concentration indicate statistically significant differences due to the interaction effect of holding time (*p* < 0.001). 1, 2: Values with different letters (in the same row) within the same holding time indicate statistically significant differences due to the interaction effect of calcium lactate concentration (*p* < 0.001).

**Table 2:** Dimensionless Shape Indicator Values of CBHB Samples.

	Dimensionless Shape Indicators		
CBHB Samples	Circularity (C)	Aspect Ratio (AR)	Sphericity Factor (SF)
C1T0	1.00	1.0	0.036
C1T30	0.99	1.00	0.042
C1T60	0.99	1.00	0.041
C2.5T0	1.00	1.00	0.061
C2.5T30	0.99	1.00	0.065
C2.5T60	1.00	1.00	0.072
C5T0	1.00	1.00	0.12
C5T30	0.99	1.00	0.16
C5T60	1.00	1.00	0.21

**Table 3:** Color properties of CBHB Samples *.

	Interaction			
	Calcium Lactate Concentration × Holding Time			
CBHB Samples	L*	a*	b*	Chroma
C1T0	51.91 ± 0.23	2.46 ± 0.51	61.76 ± 0.35	62.85 ± 0.71
C1T30	50.02 ± 1.00	2.55 ± 0.50	61.47 ± 0.12	61.52 ± 0.14
C1T60	49.24 ± 0.50	2.61 ± 0.50	61.74 ± 0.16	61.79 ± 0.20
C2.5T0	49.03 ± 0.86	4.41 ± 0.32	64.22 ± 0.34	64.36 ± 0.36
C2.5T30	48.59 ± 1.00	4.56 ± 0.37	64.48 ± 0.48	64.64 ± 0.50
C2.5T60	48.10 ± 0.41	4.71 ± 0.38	64.53 ± 0.42	64.70 ± 0.45
C5T0	48.30 ± 0.18	5.26 ± 0.83	64.90 ± 0.77	65.12 ± 0.84
C5T30	48.15 ± 0.73	5.28 ± 0.84	65.10 ± 0.55	65.32 ± 0.62
C5T60	47.82 ± 0.51	5.35 ± 0.83	65.35 ± 0.55	65.57 ± 0.86
	**Main effects**			
	**Holding time**			
	**0.**		**30.**	**60.**
L*	49.75 ± 0.74		48.92 ± 0.49	48.39 ± 0.35
a*	4.04 ± 0.59		4.13 ± 0.58	4.22 ± 0.55
b*	63.62 ± 0.65		63.68 ± 0.74	63.87 ± 0.72
**Chroma**	64.11 ± 0.52		63.83 ± 0.77	64.02 ± 0.75
	**Main effects**			
	**Calcium lactate concentration**			
	**C1**		**C2.5**	**C5**
L*	50.39 ± 0.58 ^a^		48.58 ± 0.32 ^b^	48.09 ± 0.25 ^b^
a*	2.54 ± 0.23 ^c^		4.56 ± 0.17 ^b^	5.29 ± 0.37 ^a^
b*	61.65 ± 0.12 ^b^		64.41 ± 0.20 ^a^	65.12 ± 0.29 ^a^
**Chroma**	62.05 ± 0.32 ^c^		64.57 ± 0.21 ^b^	65.33 ± 0.32 ^a^
	**Calcium lactate concentration × Holding time**		**Holding time**	**Calcium lactate concentration**
L*	ns		ns	*p* < 0.05
***a*** *	ns		ns	*p* < 0.001
b*	ns		ns	*p* < 0.001
**Chroma**	ns		ns	*p* < 0.001

* Mean values ± standard deviation; ns: not significant. a, b, c: Values with different letters (in the same line) indicate statistically significant differences due to the main effect of calcium lactate concentration (*p* < 0.001).

**Table 4:** Mechanical properties of CBHB Samples *.

	Interaction					
	Calcium Lactate Concentration × Holding Time					
CBHB Samples	Hardness (N)	Springiness (mm)	Cohesiveness (N)	Gumminess	Chewiness (Nmm)	Resilienceth
C1T0	3.49 ± 0.12 ^y3^	0.95 ± 0.04	0.80 ± 0.30	5.25 ± 0.65	3.99 ± 0.37	0.26 ± 0.01
C1T30	7.33 ± 0.15 ^x1^	0.93 ± 0.12	0.77 ± 0.07	5.16 ± 0.81	3.93 ± 0.30	0.40 ± 0.05
C1T60	7.80 ± 0.16 ^x1^	0.89 ± 0.09	0.75 ± 0.33	4.99 ± 0.23	3.16 ± 0.58	0.37 ± 0.09
C2.5T0	5.22 ± 0.11 ^y2^	0.93 ± 0.02	0.76 ± 0.03	4.25 ± 0.12	3.22 ± 0.16	0.33 ± 0.01
C2.5T30	6.59 ± 0.23 ^x1^	0.91 ± 0.04	0.70 ± 0.21	4.20 ± 0.10	3.02 ± 0.22	0.46 ± 0.05
C2.5T60	7.18 ± 0.70 ^x1^	0.88 ± 0.03	0.69 ± 0.56	4.12 ± 0.33	2.90 ± 0.18	0.47 ± 0.01
C5T0	7.53 ± 0.42 ^x1^	0.86 ± 0.25	0.72 ± 0.87	3.25 ± 0.25	3.04 ± 0.37	0.50 ± 0.06
C5T30	7.35 ± 0.21 ^x1^	0.84 ± 0.03	0.73 ± 0.03	3.23 ± 0.81	3.01 ± 0.37	0.51 ± 0.02
C5T60	7.81 ± 0.75 ^x1^	0.81 ± 0.12	0.71 ± 0.01	3.03 ± 0.23	2.82 ± 0.37	0.50 ± 0.01
	**Main effects**					
	**Holding time**					
	**Hardness**	**Springiness**	**Cohesiveness**	**Gumminess**	**Chewiness**	**Resilience**
**0.**	5.41 ± 0.15	0.91 ± 0.15	0.76 ± 0.05	4.25 ± 0.05 ^A^	3.42 ± 0.03 ^A^	0.36 ± 0.03
**30.**	7.10 ± 0.06	0.89 ± 0.05	0.73 ± 0.05	4.20 ± 0.05 ^B^	3.32 ± 0.06 ^A^	0.44 ± 0.05
**60.**	7.60 ± 0.04	0.86 ± 0.03	0.72 ± 0.06	4.05 ± 0.06 ^B^	2.96 ± 0.05 ^B^	0.45 ± 0.06
	**Main effects**					
	**Calcium lactate concentration**					
	**Hardness**	**Springiness**	**Cohesiveness**	**Gumminess**	**Chewiness**	**Resilience**
**C1**	6.21 ± 0.15	0.92 ± 0.06 ^a^	0.77 ± 0.50	5.13 ± 0.06 ^a^	3.69 ± 0.03 ^a^	0.34 ± 0.02
**C2.5**	6.33 ± 0.05	0.91 ± 0.05 ^a^	0.72 ± 0.06	4.19 ± 0.12 ^b^	3.05 ± 0.05 ^b^	0.42 ± 0.03
**C5**	7.56 ± 0.75	0.85 ± 0.09 ^b^	0.72 ± 0.09	3.17 ± 0.18 ^c^	2.96 ± 0.02 ^c^	0.50 ± 0.02
	**Hardness**	**Springiness**	**Cohesiveness**	**Gumminess**	**Chewiness**	**Resilience**
**Calcium lactate concentration × Holding time**	*p* < 0.001	ns	ns	ns	ns	ns
**Calcium lactate concentration**	ns	*p* < 0.05	ns	*p* < 0.001	*p* < 0.001	ns
**Holding time**	ns	ns	ns	*p* < 0.001	*p* < 0.001	ns

* Mean values ± standard deviation; ns: not significant. a, b, c: Values with different letters (in the same row) indicate statistically significant differences due to the main effect of calcium lactate concentration (*p* < 0.001). A, B: Values with different letters (in the same row) indicate statistically significant differences due to the main effect of post-gelation holding time (*p* < 0.001). x, y: Values with different letters (in the same row) within the same calcium lactate concentration indicate statistically significant differences due to the interaction effect of holding time (*p* < 0.001). 1, 2, 3: Values with different letters (in the same row) within the same holding time indicate statistically significant differences due to the interaction effect of calcium lactate concentration (*p* < 0.001).

**Table 5:** Formulation and Experimental Codes of CBHB Samples.

CBHB Samples	Sodium Alginate (%)	Calcium Lactate (%)	Moles Ca^2+^/g Alginate	Gelation Time (min)	Post-Preparation Holding Time (min)
C1T0	1	1	0.00459	2.5	0
C1T30		1			30
C1T60		1			60
C2.5T0		2.5	0.01147		0
C2.5T30		2.5			30
C2.5T60		2.5			60
C5T0		5	0.02294		0
C5T30		5			30
C5T60		5			60

**C1T0:** Group containing 1% calcium lactate with no post-preparation holding time; **C1T30:** Group containing 1% calcium lactate with 30 min of post-preparation holding time; **C1T60:** Group containing 1% calcium lactate with 60 min of post-preparation holding time; **C2.5T0:** Group containing 2.5% calcium lactate with no post-preparation holding time; **C2.5T30:** Group containing 2.5% calcium lactate with 30 min of post-preparation holding time; **C2.5T60:** Group containing 2.5% calcium lactate with 60 min of post-preparation holding time; **C5T0:** Group containing 5% calcium lactate with no post-preparation holding time; **C5T30:** Group containing 5% calcium lactate with 30 min of post-preparation holding time; **C5T60:** Group containing 5% calcium lactate with 60 min of post-preparation holding time.

**Table 6:** Description of dimensionless shape indicators.

Shape Indicator	Equation	Remarks
Circularity (C)	C = P^2^/4πA	The circularity varies from unity for a perfect sphere to infinity for a non-spherical object
Aspect ratio (AR)	AR = dmax/dmin	The AR varies from unity for a perfect sphere to approaching infinity for an elongated particle
Sphericity factor (SF)	SF = (dmax − dmin)/(dmax + dmin)	The deformation factor varies from 0 for a perfect sphere to approaching unity for an elongated object

P: perimeter (mm); A: area (mm^2^); dmax: largest diameter of each bead (mm); dmin: smallest diameter of each bead (mm).

## Data Availability

The raw data supporting the conclusions of this article will be made available by the authors on request.
